# Giant Left Ventricular Thrombus in a Patient with Acute Ischemic Stroke: A Case Report and Minireview

**DOI:** 10.1155/2018/3714742

**Published:** 2018-01-14

**Authors:** Fan Ye, Burton V. Silverstein, Matheen A. Khuddus, Christopher L. Bray, Arthur C. Lee

**Affiliations:** ^1^College of Medicine, University of Central Florida, Orlando, FL 32827, USA; ^2^Department of Medicine and Graduate Medical Education, North Florida Regional Medical Center, Gainesville, FL 32605, USA; ^3^North Florida Regional Medical Center, The Cardiac and Vascular Institute, Gainesville, FL 32605, USA

## Abstract

A 56-year-old healthy male with no obvious risk factors or significant past medical history was admitted to the emergency room with acute ischemic stroke. On his transthoracic echocardiography (TTE), an extremely large thrombus was detected at the apex involving the distal anterior wall. The thrombus was predominantly adherent but with a mobile tip. The patient was subsequently managed with dual antiplatelet therapy. In this report, we present an interesting case of an acute ischemic stroke secondary to a giant left ventricular thrombus in a patient with no past significant cardiac or neurologic medical history.

## 1. Introduction

Left ventricular thrombus was one of the most feared complications in patients with severe ischemic heart disease including myocardial infarction (MI) or left ventricular (LV) aneurysm [1]. Protrusion and mobility of thrombus carry a high risk for recurrent embolic strokes. The majority of reported cases of left ventricular thrombus were complications of acute MI, though it is a relatively rare complication in this day and age of expeditious revascularization but can be devastating if not diagnosed early on. However, the data on patients with large LV thrombus but without cardiac or thrombotic history are deficient.

## 2. Case Presentation

A 56-year-old Caucasian male patient with no past significant medical history or thrombotic medication use presented to the hospital with right arm weakness, slurred speech, and vertigo continuing for less than 24 hours. He had never been a smoker or illicit drug user. He was physically active and only drank socially. He had no family history of cardiovascular, pulmonary, or hypercoagulable diseases.

**Figure 1:**
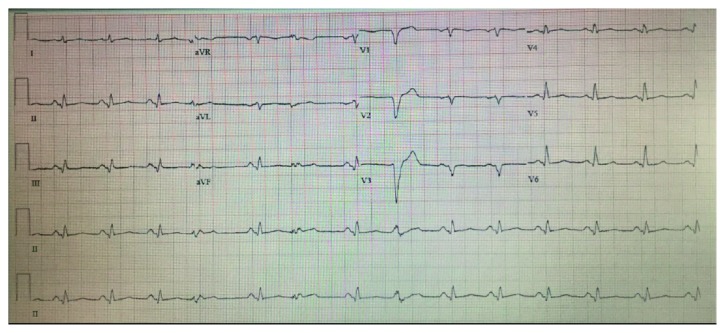
EKG at admission showing sinus rhythm with frequent premature ventricular complexes and low-voltage QRS.

**Figure 2:**
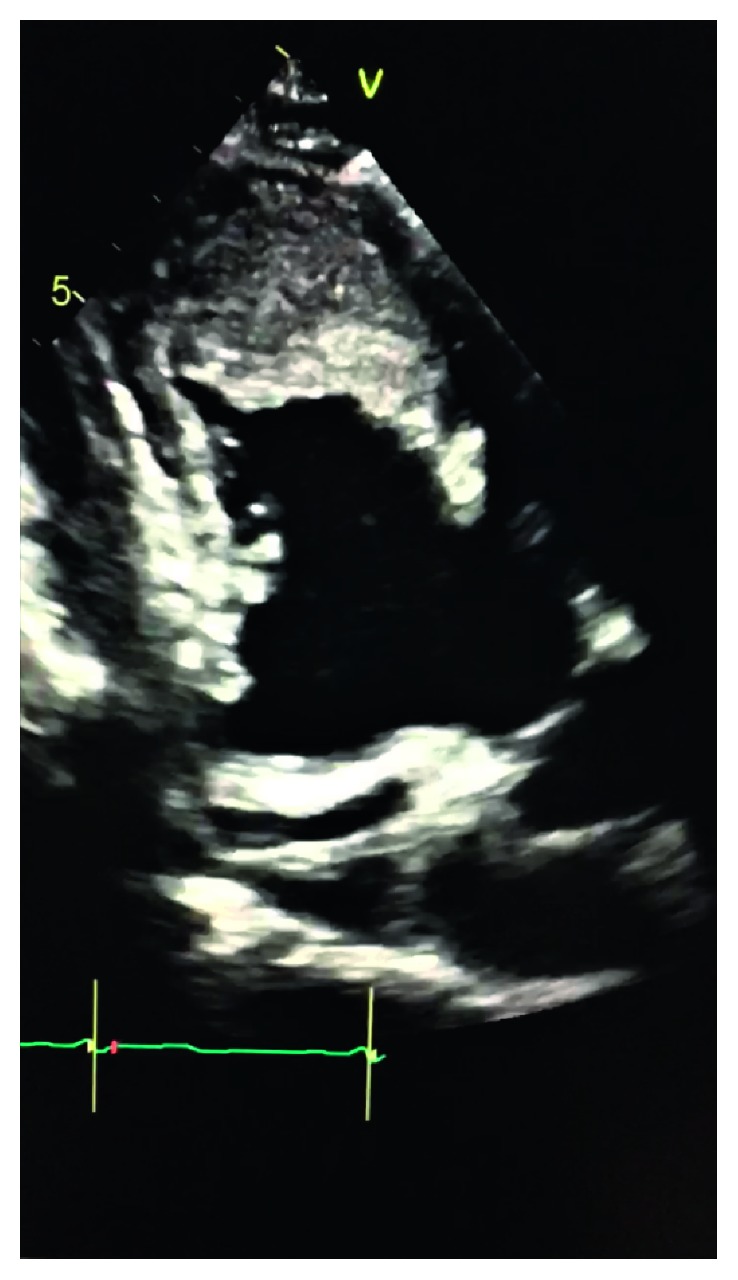
Transthoracic echocardiogram showing the left ventricle filled with thrombus.

In his initial evaluation, brain computer tomography (CT) showed acute/subacute infarct of the right posterior frontal region, brain magnetic resonance imaging (MRI) confirmed acute infarct in the left inferior frontal/suprasylvian region, and head/neck CT angiography (CTA) scan revealed no evidence for large vessel occlusion or visualized intraluminal clot. He was admitted to the neurological unit with a diagnosis of ischemic stroke and was treated with dual antiplatelet therapy using aspirin and clopidogrel. The ECG revealed sinus rhythm with frequent premature ventricular complexes and low-voltage QRS (Figure 1). The lipid panel was normal as well as kidney and liver function. Transthoracic echocardiography (TTE) detected anterior and apical wall akinesias with an LV ejection fraction (EF) of 25–30%, structurally normal valves, and an extremely large thrombus (10 cm × 12 cm) invading the apex and distal anterior wall which was predominantly adherent but with a mobile tip (Figure 2). Troponin × 3 was negative, and coagulability workup was within normal range. Subsequent myocardial perfusion imaging revealed severe hypokinesis involving the mid and apical anterior, mid anteroseptal, basal and mid inferoseptal, and basal, mid, and apical inferior wall of the left ventricle, consistent with infarction in the distribution of multiple vessels, with partial viability.

Both the cardiovascular surgeon and cardiologist agreed that the majority of the thrombus appeared densely adherent with only a small portion being somewhat mobile on echocardiography and determined the risk of myocardial resection with surgery outweighed the benefits. As a result, the patient was managed medically with aspirin 325 mg daily, clopidogrel 75 mg daily, and apixaban 5 mg bid. In addition, carvedilol 6.25 mg bid and lisinopril 10 mg daily were initiated for treatment of the ischemic cardiomyopathy. The patient was discharged three days later without any medicine side effects. We recommended a renal profile, complete blood count in three days, and TTE monthly.

## 3. Discussion

In our patient, no particular features were encountered to suggest cardiac or vascular etiology to the stroke, yet a large thrombus was detected.

The rare incidence of left ventricular thrombus has been reported in other diseases, including takotsubo cardiomyopathy [2], heparin-induced thrombocytopenia [3], Behçet disease [4], and disseminated intravascular coagulation [5]. There are limited data as to the exact frequency of LV thrombus in patients with ischemic stroke, but it has been reported to be about 2.4% in symptomatic patients by transesophageal echocardiography [6]. However, most of these studies were retrospective, nonserial, or autopsy reports. Although our patient had no significant past medical history, we speculate that his LV thrombus was secondary to his ischemic cardiomyopathy. The thrombus became firmly attached to the left ventricle, enhancing the underlying myocardial scar, limiting potential infarct expansion, and partially protecting against LV rupture. As a consequence, the patient had no symptoms until the tip of the thrombus became mobile and occluded his CNS perfusion.

Thrombi in the heart occur predominantly in males and are accompanied with ischemic or nonischemic cardiomyopathy including valvular abnormalities, arrhythmias, thromboembolism, pericarditis, and myocarditis [7]. We have recently reported that the lack of mobility is one of the key factors in most risk assessment models of thromboembolism [8]. In our patient, potential underlying myocardial ischemia caused subendothelial injury with inflammatory changes, and left ventricle regional wall akinesia resulted in blood stasis. This combination contributed to the in vivo thrombus formation [9].

The optimal management of large and pedunculated left ventricular thrombus is still controversial, especially those with low LVEF. The management is particularly challenging in patients with high bleeding risk. New oral anticoagulants (dabigatran, rivaroxaban, apixaban, etc.) have been reported in successfully treating left venous thrombus, without having limitations of vitamin K antagonists which include slow onset of action, regular monitoring, and need for dose adjustments [10]. Further evidence demonstrated that new oral anticoagulants were found to be noninferior or superior compared to warfarin in prevention of thromboembolism in patients with nonvalvular atrial fibrillation [11]. Surgical removal is generally recommended for mobile thrombi due to a significant increase in embolization risk. However, deterioration of LV function, introduction of ventricular arrhythmia, and potential dissection of the left ventricle are all serious complications related to surgery [12–14].

## 4. Conclusion

Although the reported incidence of LV thrombus which is most often seen in patients with MI has reduced significantly in the past decade with the advancement of new medications or intracardiac devices, early detection and treatment of LV thrombus in patients without past cardiac or thrombotic medical history may cause substantial variation in the reported mortality rates. Our patient had a giant LV thrombus, and his future potential risk for another systemic embolization was thought to be high. Therefore, prompt recognition and appropriate treatment of LV thrombus formation are essential to avoid deterioration of LV function and other severe complications in non-MI patients.
